# Editorial: Advances in the Multidisciplinary Management of Oral Cancer

**DOI:** 10.3389/fonc.2021.817756

**Published:** 2021-12-15

**Authors:** Alberto Paderno, Paolo Bossi, Cesare Piazza

**Affiliations:** ^1^ Unit of Otorhinolaryngology – Head and Neck Surgery, Azienda Socio Sanitaria Territoriale (ASST) Spedali Civili of Brescia, Brescia, Italy; ^2^ Department of Medical and Surgical Specialties, Radiological Sciences, and Public Health, School of Medicine, University of Brescia, Brescia, Italy; ^3^ Medical Oncology, Department of Medical and Surgical Specialties, Radiological Sciences, and Public Health University of Brescia, Azienda Socio Sanitaria Territoriale (ASST) Spedali Civili, Brescia, Italy

**Keywords:** oral cancer, multidisciplinary, management, oral cavity, squamous cell carcinoma

Oral squamous cell carcinoma (OSCC) is the 16^th^ most common neoplasm worldwide, with almost 355,000 newly diagnosed cases and over 177,000 deaths estimated in 2018 (1). Notwithstanding, it remains an often-neglected and significantly underfunded pathology, together with all neoplasms associated with stigmatized behaviors such as alcohol and tobacco consumption (2, 3). For this reason, advancements are comparatively more difficult than in more prevalent and socially accepted diseases. Furthermore, the significant incidence of high-risk behaviors in patients affected by OSCC represents an adjunctive layer of complexity in the practical application of novel achievements. Nowadays, clinicians and researchers should strive to optimize and progressively refine each aspect of OSCC management, raising awareness on its most pressing issues and selecting interventions that are applicable in everyday clinical practice.

A significant step forward in the diagnostic, therapeutic, and rehabilitative approach of OSCC (and head and neck cancers in general) has been the broad recognition of the fundamental role of multidisciplinary teams. This concept has been confirmed by the current evaluation from Shang et al., showing that patients undergoing proper multidisciplinary management had a significantly higher survival rate. This result further reinforces the need for centralization of care in OSCC, favoring institutes with the availability of a comprehensive multidisciplinary team and all the professional figures needed for the entire diagnostic and therapeutic processes, as well as post-treatment care.

As reflected by the current Research Topic, the entire patient management pathway should be optimized to obtain measurable improvements in survival and quality of life. In particular, the main fields herein addressed are tumor diagnosis and staging, surgical approaches, non-surgical treatments, and risk stratification by conventional and molecular techniques.

Proper and timely diagnosis remains a weak point in management of OSCC. Tumors are often referred to specialists with significant delay and after reaching advanced stages. This factor has a significant impact on prognosis and is the first variable that should be optimized. Bioendoscopic filters such as Narrow Band Imaging (NBI) can improve the diagnostic potential of conventional oral examination (4, 5). However, these techniques require a significant learning curve and are burdened by subjectivity in interpretation. The study by Paderno et al. showed for the first time the possibility to apply fully convolutional neural networks to NBI endoscopic frames of oral lesions in order to automatically identify tumors and delineate their margins. This preliminary report confirms the potential of the newly developing field of “Videomics” for diagnosis and in-depth characterization of OSCC (6). In parallel, efforts should be made to refine diagnosis and stratification of oral potentially malignant diseases, thus identifying those at higher risk of malignant transformation. These two fields of research, i.e. the definition of high-risk premalignant lesions and early identification of OSCC, have a lot of common ground, being part of the continuum process of cancerization.

When considering OSCC treatment, surgery still remains the first-line option, potentially followed by adjuvant therapies. However, surgery is not a static discipline, and techniques should be refined and evolve according to new evidence and technologies. In recent years, the concept of compartmental surgery for OSCC has gained significant momentum (7–9). In this regard, Carta et al. and Grammatica et al., respectively, provided a retrospective analysis confirming the good oncologic outcomes obtainable by compartmental tongue resections and a step-by-step guide describing such a surgical technique.

At the same time, the growing acceptance of sentinel lymph node biopsy in oral oncology may lead to improvements in prophylactic management of contralateral neck metastases. As Mahieu et al. reported, the contralateral neck is generally not addressed by elective neck dissection in early stage OSCC not involving the midline, while sentinel lymph node biopsy may stage both the ipsilateral and contralateral neck. Interestingly, the authors described a higher rate of contralateral regional recurrence in patients receiving elective neck dissection than those who underwent sentinel lymph node biopsy. This result shows the effectiveness of such a procedure in detecting unexpected contralateral nodal spread, possibly opening new applications for this technique in the setting of minimally invasive contralateral neck staging.

However, optimization of surgical treatment is not limited to the simple therapeutic stage. van Baar et al., in fact, assessed a novel treatment concept for advanced mandibular osteoradionecrosis combining isodose curve visualization and alveolar nerve preservation. This pilot study showed the promise of three-dimensional planning in mandibular resection and reconstruction, taking into account previous radiotherapy fields and maximizing sensory preservation. In adjunction, non-surgical therapies have also been assessed, given the progressive improvements of radiation techniques and chemotherapy regimens. Kim et al. compared postoperative chemoradiation with radiotherapy alone using new generation techniques, showing comparable results except for tumors with extranodal extension. Different schedules of induction chemotherapy have been presented, attesting the better tolerability of weekly induction taxane – platinum – fluorouracil in comparison to a 3-week schedule (Tousif et al.). These results should, however, be put within the context of the lack of survival benefit obtained with induction chemotherapy in OCSCC. Looking to drug repurposing, a potential synergic effect has been found when low molecular weight heparin is added to cisplatin (Camacho-Alonso et al.). Still, drug discovery may conceivably offer novel tools for treatment of OSCC. In this regard, melatonin can exert anti-proliferative, anti-invasive, and anti-migrative effects on OSCC *via* the miR-25-5p/NEDD9 pathway, thus warranting further assessment of its potential (Wang et al.).

However, alternative treatments in inoperable and oligometastatic OSCC should also be explored. Lambert et al. reported a single-center experience on the use of photodynamic therapy as an alternative treatment tool in inoperable oral and oropharyngeal cancer. While limited to highly selected patients, functional and oncologic outcomes were satisfying considering the specific setting. Swallowing and airway patency were preserved in 77% and 96% of patients, respectively, and the recurrence-free rate at two years was 32%.

Furthermore, according to Szturz et al., mounting evidence suggests that patients with few and slowly progressive distant metastases of small size may benefit from various local ablation techniques. The authors summarize the potential of surgery and stereotactic radiotherapy in this specific setting. In particular, patients presenting with late development of slowly progressive oligometastatic lesions in the lungs are deemed to be potential candidates for metastasectomy or other local therapies. While literature data is still limited, this review highlights and carefully describes the often-neglected issue of oligometastatic disease in head and neck cancer, where the areas of research will increase in the future, thanks to the exploitation of combinations with immunotherapy.

Of note, significant effort has been directed towards predicting prognosis and treatment response in OSCC, resulting in improved patient stratification. This possibility has been explored through various methods, starting from conventional risk-factor selection to the evaluation of gene expression and genomic signatures.

As demonstrated, the clinical and pathologic features can be effectively integrated to optimize risk stratification through prognostic scoring systems (Zhou et al.). This is the first step towards treatment personalization, an ever-growing trend in modern oncology that aims to refine patient management by carefully assessing individual characteristics. However, accurate in-depth disease modeling requires considering a wide variety of variables that go beyond conventional clinical and histopathological characteristics. The overall microenvironment and immune-context have a leading role in determining tumor initiation, progression, and clinical features. The oral microbiota is an adjunctive player that adds complexity to the intricate web of tumor-host relations, and dysbiosis of the oral microbiome was also shown to play a critical role in the initiation and progression of OSCC. Here, Sarkar et al. precisely described the changes in the oral microbial community in Indian patients, giving a point of reference for future assessments. Considering the analysis of the tumor-immune system interplay, Fan et al. investigated the role of frequency of heterotypic neutrophil-in-tumor structure for the prognosis of patients with OSCC, highlighting its independent association with both recurrence-free and disease-specific survivals. Finally, diving into deeper and finer characteristics, gene expression profiling is showing significant promise in assessing and stratifying OSCC. In particular, mitochondrial serine hydroxymethyltransferase overexpression appears to correlate with advanced pathological grade and recurrence (Wu Z-Z et al.). In addition, interleukin 1 receptor associated kinase 2 (IRAK2) overexpression is associated with enhanced radiosensitivity of OSCC cells, and tumors with high IRAK2 expression had better post-irradiation local control than those with low expression (Yu et al.). However, while targeted evaluations of single-gene expression may be helpful to fully clarify mechanisms and potential as therapeutic targets, complete transcriptomic analysis may give a comprehensive view of tumor biologic characteristics and risk profile. In this regard, Wu X et al. developed a 5-metabolic pathway prognostic signature for OSCC based on dysregulated metabolic cascades and provides support for the aberrant metabolism underlying tumorigenesis. The signature effectively stratified patients in groups according to survival and served as an independent prognostic factor.

Finally, the vast majority of tumors in the oral cavity are definitively squamous cell carcinoma. Nevertheless, while significantly less prevalent, salivary gland cancers also warrant consideration. Park et al. provided an overall view of minor salivary gland cancers originating from the oral cavity and compared them with OSCC. Interestingly, patients with small salivary gland malignancies showed >90% survival at 5 years, and local control was often successful even with close or positive margins. However, treatment choice should still take into account the vast heterogeneity of biological behavior in salivary gland tumors. In fact, different subtypes, even if defined by the same histology (Piwowarczyk et al.), may present diverse growth rates, patterns of spreading, and likelihood of recurrence.

In conclusion, the management of OSCC has significant room for improvement, and this should be primarily obtained by optimizing current strategies. Indeed, many factors that decrease survival are related to late diagnosis or inadequate treatment and could be addressed by prompt referral to leading oncologic centers. Once this issue has been solved, the introduction of molecular analyses and artificial intelligence tools have the potential to further improve treatment personalization and outcomes.

## Author Contributions

AP, PB, and CP wrote the manuscript, contributed to manuscript revision, read, and approved the submitted version. 

## Conflict of Interest

The authors declare that the research was conducted in the absence of any commercial or financial relationships that could be construed as a potential conflict of interest.

## Publisher’s Note

All claims expressed in this article are solely those of the authors and do not necessarily represent those of their affiliated organizations, or those of the publisher, the editors and the reviewers. Any product that may be evaluated in this article, or claim that may be made by its manufacturer, is not guaranteed or endorsed by the publisher.
